# Noninvasive DWI tracking of hiPSCs differentiation into RTECs in AKI recovery via the KSP promoter-mediated AQP1 strategy

**DOI:** 10.7150/thno.109826

**Published:** 2025-04-09

**Authors:** Yue Zhao, Mingfu Gong, Xu Liu, Qian Xie, Tao Sun, Chunyu Zhou, Tongsheng Shu, Shuang Wang, Liang Zhang, Dong Zhang

**Affiliations:** Department of Radiology, Xinqiao Hospital, Army Medical University, Chongqing 400037, China.

**Keywords:** human-induced pluripotent stem cells, acute kidney injury, aquaporin 1, diffusion-weighted imaging, tracking of differentiation

## Abstract

**Rationale:** Human-induced pluripotent stem cells (hiPSCs) exhibit great potential in the treatment of acute kidney injury (AKI), and their targeted differentiation into renal tubular epithelial cells (RTECs) is directly involved in the repair of the injured tissue. However, their differentiation during treatment is difficult to evaluate noninvasively.

**Methods:** Aquaporin 1 (AQP1) can alter the dispersion of water molecules at the cellular level and thus can be detected with high sensitivity by diffusion-weighted imaging (DWI). In this study, a kidney-specific promoter (KSP-cadherin)-driven AQP1 overexpression lentivirus (KSP-AQP1) was constructed and used for tracking the differentiation of hiPSCs *in vivo*. Then, two AKI animal models were used to identify the feasibility of KSP-AQP1 for *in vivo* tracking of the differentiation of hiPSCs into RTECs.

**Results:** We utilized KSP-positive and KSP-negative cells to examine the *in vitro* specificity of KSP-AQP1. It was found that only the KSP-positive cells showed a substantial expression of AQP1, accompanied by a significant variation in both the diffusion-weighted imaging (DWI) signal intensity (SI) and the apparent diffusion coefficient (ADC) values. whereas KSP-AQP1-transduced KSP-negative cells had no apparent SI and ADC changes. DWI results suggested that after the hiPSCs transplantation *in vivo*, the KSP-AQP1-pretransduced hiPSCs group exhibited a significantly decreased SI and increased ADC value when compared with the hiPSCs-treated and untreated AKI kidneys. In addition, the AQP1-mediated differences in DWI SI and ADC value between the KSP-AQP1-pretransduced hiPSCs group and hiPSCs group were confirmed by analysis of the KSP transcriptional activity using co-expressed exogenous flag gene mCherry.

**Conclusions:** This study successfully developed a method for tracking the differentiation of hiPSCs into RTECs *in vivo* during the treatment of AKI using a KSP-regulated AQP1 overexpression strategy.

## Introduction

Acute kidney injury (AKI) is a common and seriously adverse clinical condition indicated by apoptosis and necrosis of renal tubular epithelial cells (RTECs), with an incidence rate of 5-10% in hospitalized patients and as high as 30-50% in an intensive care unit 1, 2. Currently, the treatment for AKI includes drug therapy, renal replacement, and supportive therapies. However, their efficacy in restoring injured renal tissues is limited 3, 4. Recently, stem cell-based therapy has emerged as a promising method for AKI treatment due to its outstanding self-renewal and pluripotent differentiation potential 5, 6. Particularly, human-induced pluripotent stem cells (hiPSCs) can differentiate directly into RTECs, thus recovering the damaged renal tubular epithelial tissue 7, 8. And the more target differentiation of hiPSCs into RTECs, the better outcome of the treatment. However, the differentiation process of hiPSCs *in vivo* is complex and varies along with the individualized damaged microenvironment. Therefore, given the crucial role in reflecting the effectiveness of the treatment, monitoring the differentiation process becomes of utmost importance.

Fluorescence-based imaging has been extensively used for *in vitro* cell tracking 9, 10. However, its application in *in vivo* cell tracking has been significantly constrained because of limited penetration depth and tissue autofluorescence 11-13. Although computed tomography and positron emission tomography-computed tomography are unaffected by tissue depth, their radiative properties render them unsuitable for continuous *in vivo* tracking 14-16. Conversely, magnetic resonance imaging (MRI) possesses several advantages, including high resolution, non-radiative nature, deep tissue penetration, and minimal effect on cellular physiological functions 17, 18. Molecular imaging techniques based on MRI enable qualitative and quantitative analyses of the cellular or even molecular level *in vivo* when specific molecular probes are used 19. Nevertheless, among various MRI molecular probes, it remains challenging to modulate MRI signal transformations within cells for the tracking of differentiation after labeling with exogenous MRI contrast agents, such as magnetic nanoparticles and gadolinium 20. Furthermore, as cells divide, the concentration of these exogenous contrast agents per cell gradually reduces 21, limiting their use in differentiation tracking. In contrast, MRI reporter gene patterns are a very promising candidate for long-term tracking with the help of gene expression modulation strategies 22. To date, the development of various MRI reporter gene systems includes metal-associated reporter genes expressing ferritin and transferrin (i.e., iron-rich proteins) and reporter genes generating intrinsic contrast through the chemical exchange saturation transfer effect 23. However, the former call for consideration concerning biocompatibility and bioavailability for that metal, whereas ferritin-based reporter gene imaging will need additional iron. The chemical exchange saturation transfer-based reporter gene system has a low sensitivity and frequently requires higher levels of gene expression for detectable MRI contrast, proving that it is not very practical 24-26.

Aquaporin 1 (AQP1) is a transmembrane protein localized on the cell membrane that can facilitate the transport of water molecules across the cytomembrane, and play a key role in regulating the balance of water between intracellular and extracellular environments 27, 28. The intracellular-to-extracellular water exchange rate can be determined through shutter-speed analysis of Dynamic Contrast Enhanced-MRI and Filter-Exchange imaging. However, the former necessitates the use of exogenous contrast agents, such as manganese ions, to reach the target cell interstitium, which introduces certain limitations *in vivo* 29. Additionally, Filter-Exchange imaging requires specialized sequences and post-processing software, and the lack of a standardized parameter protocol results in low data comparability across different centers 30. Alternatively, diffusion-weighted imaging (DWI) has reached clinical maturity and benefits from standardized protocols. Numerous studies have demonstrated the feasibility of correlating AQP1 overexpression with the local apparent diffusion coefficient (ADC) and the signal intensity (SI) of DWI 31-33. Using a high *b*-value DWI sequence, it can detect the diffusion characteristics of water molecules, thereby reflecting the expression levels of AQP1 on the membrane indirectly. In addition, previous researches have indicated that the overexpression of AQP1 shows no potential toxicity on cells, and an increase of 10% in AQP1 level can generate significant DWI contrast 34-36.

To regulate the expression of exogenous MRI reporter genes controllable, an upstream control element-gene promoter-should be considered simultaneously. Compared with inducible gene promoters, such as the doxycycline-regulated Tet-On system, tissue-specific promoters offer the key advantage of not requiring additional human intervention. These tissue-specific promoters are exclusively activated within specific cell types, thereby demonstrating enhanced feasibility for *in vivo* cell tracking applications 37, 38. Previous researches have shown that the genetic characteristics of hiPSCs change in accordance with their differentiation. Specifically, for stem cell-based treatment of AKI, the phenotypic transitions from hiPSCs into RTECs will lead to the selective activation of the kidney-specific promoter (KSP-cadherin) 39-41.

Here, this study attempted to develop an MRI reporter lentivirus system (KSP-AQP1) using the KSP promoter and AQP1 gene, which was previously introduced into hiPSCs as a differentiation tracking system. After assessing the correlation between AQP1 expression levels and DWI SI and ADC values *in vitro*, the hiPSCs engineered with the KSP-AQP1 were used for AKI treatment, and DWI was used for tracking the differentiation *in vivo*.

## Methods

### Cell culture

hiPSCs were cultured in ncTarget hPSC medium (Shownin, Anhui, China) supplemented with Matrigel (ABW, Shanghai, China). The HK-2 and AML12 cells were cultured in Dulbecco's modified Eagle medium supplemented with 10% fetal bovine serum, 100 µg/mL streptomycin, and 100 U/mL penicillin. All cultures were incubated at 37 °C in a 5% CO_2_ atmosphere (Thermo, Waltham, MA, USA).

### Lentivirus preparation and cells transduction

A CV130 backbone vector incorporating the red fluorescent protein gene mCherry was used to construct the recombinant lentiviral plasmid. A DNA fragment containing the KSP sequences and AQP1 coding sequence was designed with restriction endonuclease sites of *Pac*I and *Eco*RI at each terminus and synthesized by Shanghai GeneChem. Subsequently, the backbone vector was also digested with the same restriction enzymes, *Pac*I and *Eco*RI, and ligated with the designed DNA fragment using T4 DNA ligase (Figure S1). After the insertion of the DNA fragment, the plasmid was confirmed by plasmid polymerase chain reaction (PCR) and DNA sequencing, and then used for lentivirus packaging. The titer of the packed lentivirus was identified using PCR (GeneChem, Shanghai, China). The resulting lentivirus was named KSP-AQP1, and the morphology of KSP-AQP1 was observed using transmission electron microscopy. All prepared lentiviruses were sub-packed and stored at -80 °C until further use.

To transduce the KSP-AQP1 into cells, the HK-2, AML12, and hiPSCs were cultured under standard cell culture conditions and subsequently harvested after trypsinization. Then approximately 5,000 cells were seeded into the well of a 24 well plate and incubated at 37 °C overnight. The next day, the culture medium was replaced with fresh medium containing KSP-AQP1 at a multiplicity of infection of 10 and 1 × HiTransG P. Then, the cells were further incubated at 37 °C for another 12 h. Subsequently, the medium was removed and replaced with 1 mL fresh medium and continued for an additional 72 h to induce the expression. The expression of KSP-AQP1 was visualized using an inverted fluorescence microscope (Leica Microsystems, Wetzlar, Germany). The hiPSCs transduced with KSP-AQP1 were named lenti-hiPSCs.

### Differentiation of hiPSCs

The differentiation of hiPSCs and lenti-hiPSCs was performed using the Nephron Differentiation Kit (Shownin, Anhui, China). A 3-day culture with ncTarget hPSC medium supplemented with Matrigel was performed until approximately 50% confluence. The cells were then washed once with Dulbecco's Phosphate Buffered Saline, and the medium was replaced with differentiation medium A (day 0). The growth medium was thus changed once a day with fresh medium A for 3 days. On day 4, the differentiation medium was changed to medium B, which was also changed daily for the next 2 days. On day 6, the culture medium was changed to complete differentiation medium C for the next 2 days with daily medium changes. On day 8, the cells were switched to complete differentiation medium D for another 2 days with daily medium change. On day 10, the medium was reverted to complete differentiation medium C, and the cells were kept for an additional 2 days with daily medium changes. From day 13 to day 20 of differentiation, the culture medium was switched to medium E daily. After the supplementation of culture medium E, the mature renal epithelial cells (named hiPSC-RTECs) would typically appear for approximately 5-7 days. The renal epithelial cells were preserved and characterized on day 20 of differentiation and later subjected to further experimentation. The RTECs derived from the differentiation of lenti-hiPSCs were named lenti-hiPSC-RTECs. For animal experiments involving hiPSCs, the initial phase of differentiation is pivotal for facilitating the desired differentiation outcomes 42, 43. By allocating approximately 6 days of differentiation for hiPSCs and lenti-hiPSCs (named diff-hiPSCs and diff-lenti-hiPSCs, respectively), these cells become more suitable for subsequent experimental procedures in animals.

### mRNA expression analysis

Total RNA was prepared from the cells using the BGMG Fast Total RNA Extraction Kit (Baoguang, Chongqing, China), and then genomic DNA was removed from the extracted total RNA, and complementary DNA was synthesized by the PrimeScript™ FAST RT Reagent Kit (Takara, Dalian, China) with gDNA Eraser. The real-time reverse transcription-PCR (RT-PCR) assay was performed on an ABI 7500 using the TB Green^®^ Premix Ex Taq™ II FAST qPCR (Takara, Dalian, China) reagent. The primers used in this study are described in **Table S1**. For data analysis, the 2^-ΔΔCt^ method was used for calculating the relative mRNA expression, which was normalized to GAPDH.

### Western blot analysis

First, total proteins were extracted using a total protein extraction kit (Baoguang, Chongqing, China), following the manufacturer's protocol. Subsequently, the concentrations of the obtained proteins were determined using a BCA protein quantification kit (Beyotime, Shanghai, China). Then, approximately 20 μg of total protein from each sample was mixed with loading buffer, subjected to heat denaturation, and loaded into the wells of an SDS-PAGE gel (Servicebio, Wuhan, Hubei, China) for electrophoretic separation. Following separation, the proteins were transferred onto a PVDF membrane. Then, 5% bovine serum albumin was used to block the nonspecific binding sites on the membrane. Thereafter, the membrane was incubated overnight at 4 °C with primary antibodies targeting OCT4 (1:1000, Proteintech, Wuhan, Hubei, China), CDH1 (1:500, Servicebio, Wuhan, Hubei, China), AQP1 (1:1000, Proteintech, Wuhan, Hubei, China), and β-actin (1:1,000, Servicebio, Wuhan, Hubei, China). Then, a secondary goat anti-rabbit or anti-mouse IgG antibody (1:3,000, Solarbio, Beijing, China) conjugated with horseradish peroxidase was used for another 1 h incubation at room temperature. Finally, after washing with TBST three times, each for 10 min, the proteins were visualized using an Enhanced Chemiluminescence Kit (Beyotime, Shanghai, China) and the ImageQuant LAS 4000 (GE Healthcare Life Sciences, USA). The relative expression levels of the proteins were assessed by quantifying the grayscale values of the target proteins in comparison with β-actin.

### Immunofluorescence staining assay

Immunofluorescence staining assay (IFA) was performed to detect the target gene expression. In brief, the cells were first fixed with 4% paraformaldehyde. Then permeabilization was performed by 1% Triton X-100, followed by blocking with 5% bovine serum albumin for 1 h. Subsequently, the cells were incubated at 4 °C overnight with different target primary antibodies, including OCT4 (Proteintech, Wuhan, Hubei, China), CDH1 (Servicebio, Wuhan, Hubei, China), and AQP1 (Proteintech, Wuhan, Hubei, China). Then, fluorescent-labeled secondary antibodies (Servicebio, Wuhan, Hubei, China), including AF488-labeled goat anti-rabbit IgG, AF594-labeled goat anti-mouse IgG, and AF594-labeled goat anti-rabbit IgG, were added and co-incubated with the cells for another 1 h in dark. The cell nucleus was stained with DAPI, and the stained cells were observed under an inverted fluorescence microscope.

### Cell MRI

For cell MRI, the hiPSCs, lenti-hiPSCs, hiPSC-RTECs, and lenti-hiPSC-RTECs were initially washed with Phosphate Buffered Saline (PBS) to eliminate dead cells. Following this, the cells were harvested through trypsin digestion and centrifugation (300g for 5 minutes) to get a cell pellet. The pellets were then embedded in four Eppendorf tubes and pre-equilibrated to a temperature of 22 ± 0.5°C. These preparations were subsequently placed onto a 1% agarose platform to construct a cell model compatible with the MRI coil. Three MRI scannings were conducted, with the sample order being altered for each scanning. MRI scanning was performed using a 7.0-T MRI scanner (Bruker, Ettlingen, Germany) equipped with a transceiver coil featuring a 7.2 cm diameter aperture. The DWI parameters were as follows: repetition time (TR) = 2.5 s, echo time (TE) = 24.5 ms, flip angle (FA) = 90°, number of excitations (NEX) = 2, matrix size = 256 × 256, field of view (FOV) = 4.0 × 3.5 cm^2^, slice thickness/spacing = 0.5 mm/0.5 mm, and *b*-values = 0, 450, 650, 800, 1,000, 1500, and 2000 s/mm^2^. The T_2_-weighted imaging (T_2_WI) parameters were as follows: TR = 2.5 s, TE = 24 ms, FA = 90°, NEX = 2, matrix size = 256 × 256, FOV = 4.0 × 3.5 cm^2^, and slice thickness/spacing = 0.5 mm/0.5 mm. For each group, a circular region of interest (ROI) was delineated to encompass the entire cell suspension, maximizing the diameter. Each measurement was conducted independently and repeated three times. The ADC value was calculated according to the formula ADC= Ln(*S*_2_/*S*_1_)/(*b*_1_ - *b*_2_). The ImageJ software (NIH) was used to analyze the SI of DWI and T_2_WI images.

### Animal models

The animal protocol of this experiment was reviewed by the Laboratory Animal Ethics Committee of Army Medical University (No. AMUWEC20235196). The animal experiment was strictly performed in accordance with the National Institutes of Health Guide for the Care and Use of Laboratory Animals to safeguard the welfare of the experimental animals and the scientific, standardized, and ethical rationality of the experiment.

SPF grade, 4-6-week-old, male Sprague-Dawley rats were purchased from Beijing Huafukang Biotechnology (Beijing, China). Two AKI models were used in this research: ischemia/reperfusion (I/R) and cisplatin. For each model, the kidneys were allocated into four groups: the control kidney, the AKI kidney, the AKI with diff-hiPSCs kidney, and the AKI with diff-lenti-hiPSCs kidneys.

To prepare the two AKI models, the following methods were coded. Bilateral kidney I/R model: Upon the anesthetization of rats by isoflurane application, incisions were made through the midline of the abdomen to expose both kidneys, and the bilateral renal arteries were clamped for 1 h. The kidney's ischemic state was confirmed by observing the whitening of the kidney. Afterward, the arterial clamp was removed carefully, and kidney reperfusion was confirmed with the naked eye within 5 min, then the left renal artery was injected with either 1 × 10⁶ diff-hiPSCs or diff-lenti-hiPSCs as treatment groups, while the control got normal saline. The syringe was left in place for ~5 min before removal, and then skin sutures completed the surgery. For the cisplatin-induced AKI model, rats received an intraperitoneal injection of 20 mg/kg cisplatin. One hour later, under anesthesia, a mid-line abdominal incision exposed the left kidney. Two treatment groups were set up: one had 1 × 10⁶ diff-hiPSCs and the other 1 × 10⁶ diff-lenti-hiPSCs injected into the left renal artery. After injection, the syringe was left in place for ~5 min before removal, and then skin sutures finished the surgery.

### Animal MRI

In the animal magnetic resonance experiment, MRI scans were performed before modeling and on the 7th and 14th days after cell transplantation. First, the rats were anesthetized and then placed on the Philips MR Systems Ingenia 3.0 T magnetic resonance equipment to undergo DWI and T_2_WI. The DWI scanning parameters were as follows: TR = 5 s, TE = 82 ms, FA = 90°, NEX = 2, matrix size = 256 × 256, FOV = 3.5 × 3.5 cm^2^, slice thickness/slice interval = 1.0 mm/1.0 mm, and *b*-values = 0, 450, 650, and 1,000 s/mm^2^. The T_2_WI scanning parameters were as follows: TR = 2.5 s, TE = 80 ms, FA = 90°, NEX = 2, matrix size = 256 × 256, FOV = 4.0 × 4.0 cm^2^, and slice thickness/slice interval = 1.0 mm/1.0 mm. After the scanning was completed, the Philips post-processing software was used to analyze the results of DWI and T_2_WI images. The ROI was defined on the renal parenchyma using T_2_WI and subsequently transferred to the DWI image, as illustrated in Figure S2.

### Renal function and pathological analysis

To assess the therapeutic effects of hiPSCs in AKI, serum renal function and renal tissue histological analyses, including H&E, Immunohistochemical (IHC) and Masson's trichrome staining, were performed. Blood samples were collected from the tail vein before AKI induction and on the 7th and 14th days following cell transplantation. Serum was isolated by centrifugation, and the concentrations of blood urea nitrogen (BUN) and serum creatinine (SCr) were detected using an automated biochemical analyzer. For histological evaluation, kidney tissues were harvested from the rats, fixed in 4% paraformaldehyde, and subsequently embedded in paraffin for sectioning and staining. The cell density, size and morphology of the renal tissue sections were examined microscopically, and the tubular injury scoring was conducted according to the tubular injury scoring criteria (Extend Method in Supplementary Material).

### Statistical analysis

Statistical analysis was performed using GraphPad Prism 9.5 (GraphPad Software, USA). The results are expressed as mean ± standard error. Student's t test was used for two-group comparisons, and Dunnett's multiple comparison test was used for a one-way analysis of variance for three or more groups to analyze the significance of differences. *p < 0.05; **p < 0.01; ***p < 0.001; ****p < 0.0001.

## Results

### Preparation and specificity of lentiviral KSP-AQP1

**Figure 1:**
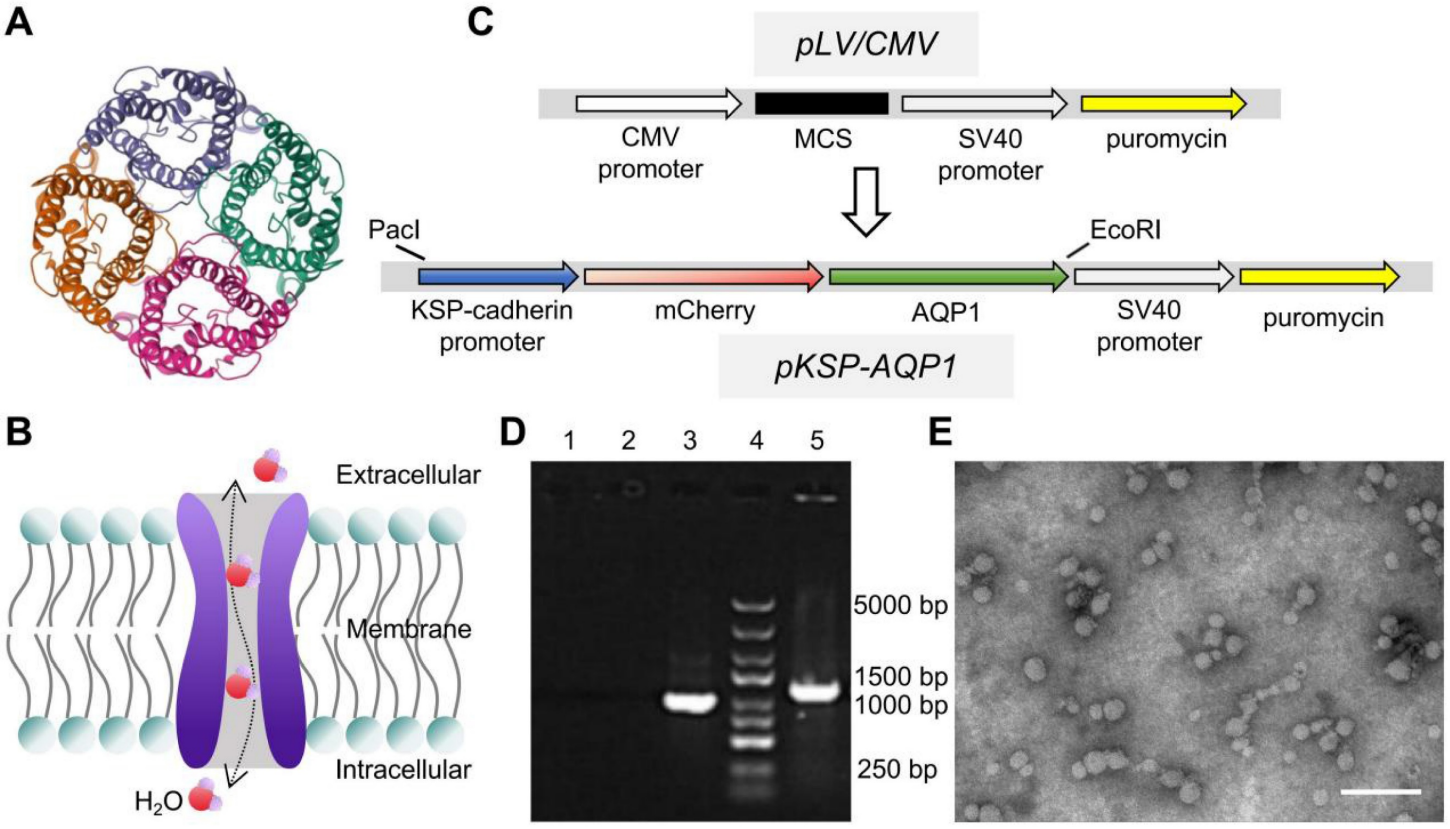
** AQP1 structure and lentiviral construction.** (**A**) The 3D structure information of AQP1 (PDB ID: 1ih5). (**B**) Schematic illustration of the transportation process of water molecules within the AQP1 channel. (**C**) The workflow of construction of recombinant lentivirus plasmids. (**D**) PCR analysis of the plasmids. 1, ddH_2_O; 2, *pLV/CMV* plasmid; 3, GAPDH; 4, DNA marker; and 5, *pKSP-AQP1* plasmid. (**E**) Transmission electron microscopic observation of KSP-AQP1. Scale bar, 200 nm.

AQP1 is a channel-like integral membrane protein that selectively transports water molecules without the consumption of adenosine triphosphate (**Figure 1A-B**) 44, 45. A lentiviral system, regulated by the KSP promoter to drive the overexpression of the AQP1 protein, was prepared as a tool to induce AQP1 overexpression in RTECs. In addition, the gene encoding the red fluorescent protein mCherry was also incorporated as a visual marker for expression (**Figure 1C**). The engineered lentiviral plasmid was initially identified using PCR, which detected a specific fragment within the recombinant plasmid containing sequences of AQP1 and vector (**Figure 1D**). Gene sequencing was also used here to confirm the accuracy of the sequences of AQP1 gene and KSP-cadherin promoter (**Supplementary File 1**). The prepared lentivirus was observed using TEM, which exhibited a spherical morphology with a diameter of 30-60 nm (**Figure 1E**).

### Differentiation of hiPSCs into RTECs

**Figure 2:**
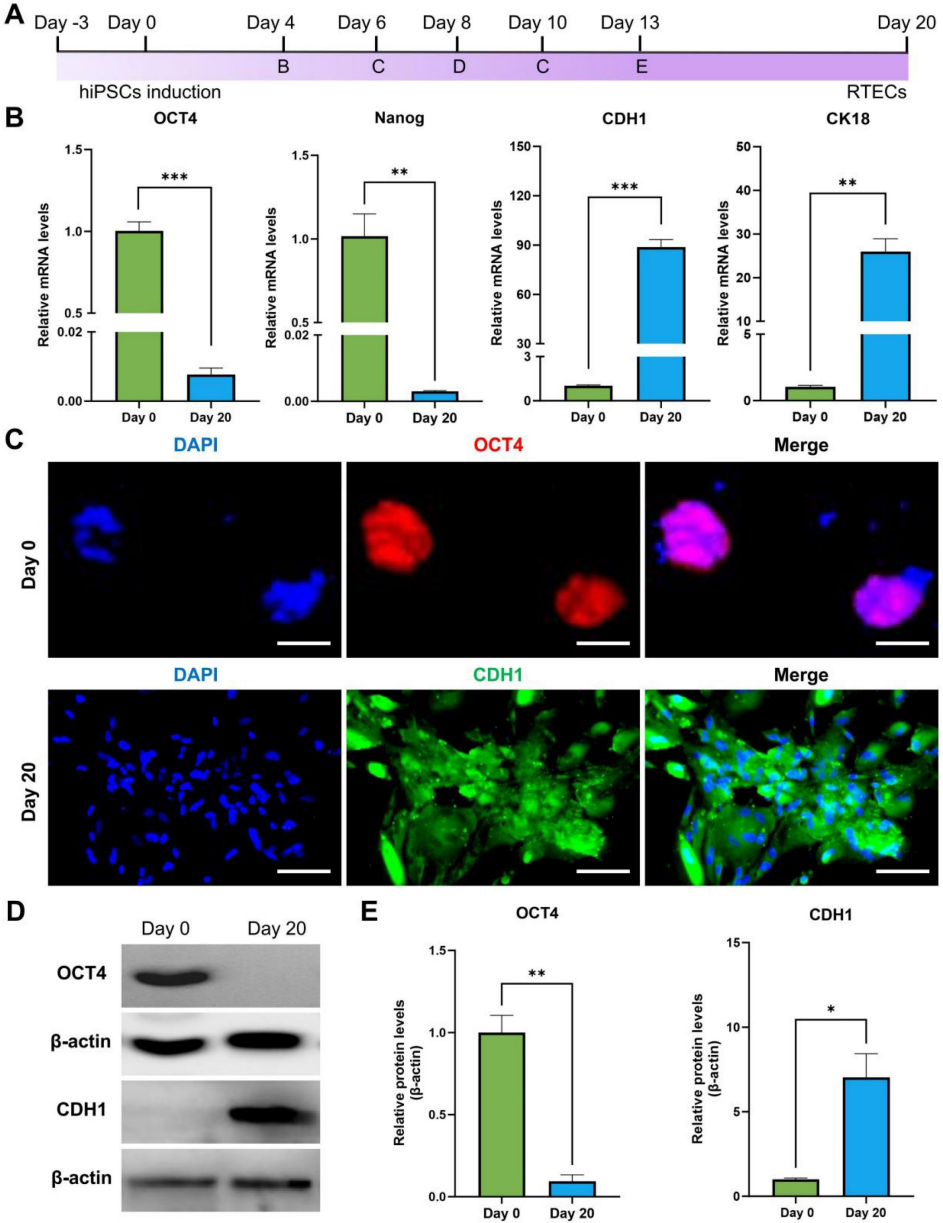
** Differentiation of hiPSCs into RTECs.** (**A**) Schematic of the differentiation process using the Nephron Differentiation Kit. (**B**) RT-PCR analysis revealed significant expression of pluripotency markers OCT4 and Nanog on Day 0 and epithelial cell markers CDH1 and CK18 on Day 20. (**C**) IFA indicated a significantly high expression of the pluripotency marker OCT4 on Day 0 and the epithelial cell marker CDH1 on Day 20. Scale bar, 100 μm. (**D**) Western blot assay demonstrated significantly high expression of the pluripotency marker OCT4 on Day 0 and the epithelial cell marker CDH1 on Day 20. (**E**) Quantitative analysis of western blot results. *p < 0.05, **p < 0.01, ***p < 0.001; n = 3.

Differentiation of hiPSCs was performed following the workflow presented in **Figure 2A**. The hiPSCs markers (OCT4 and Nanog) and RTECs markers (CDH1 and CK18) were analyzed before and 20 days post-differentiation to confirm the success of differentiation. As presented in **Figure 2B**, RT-PCR results suggested that the relative mRNA expression levels of OCT4 and Nanog were significantly elevated on Day 0, but CDH1 and CK18 exhibited significantly high levels on Day 20 of differentiation. In addition, the expression levels of OCT4 and CDH1 were also analyzed by IFA. As presented in **Figure 2C**, OCT4 was stained with the Alexa Fluor 594-labeled secondary antibody and exhibited bright red fluorescence, representing a high level of OCT4 in hiPSCs. For CDH1, the Alexa Fluor 488-labeled secondary antibody was used, resulting in bright green fluorescence in the cells that had undergone 20 days of differentiation, indicating a higher expression of CDH1. Furthermore, the expression of OCT4 and CDH1 was identified using western blot (**Figure 2D-E**), demonstrating a similar expression tendency with RT-PCR and IFA. The results demonstrated that the hiPSCs maintained a good stemness prior differentiation-induction. After 20 days of induction, these cells were successfully differentiated into RTECs.

### KSP-AQP1-mediated AQP1 overexpression in RTECs

**Figure 3:**
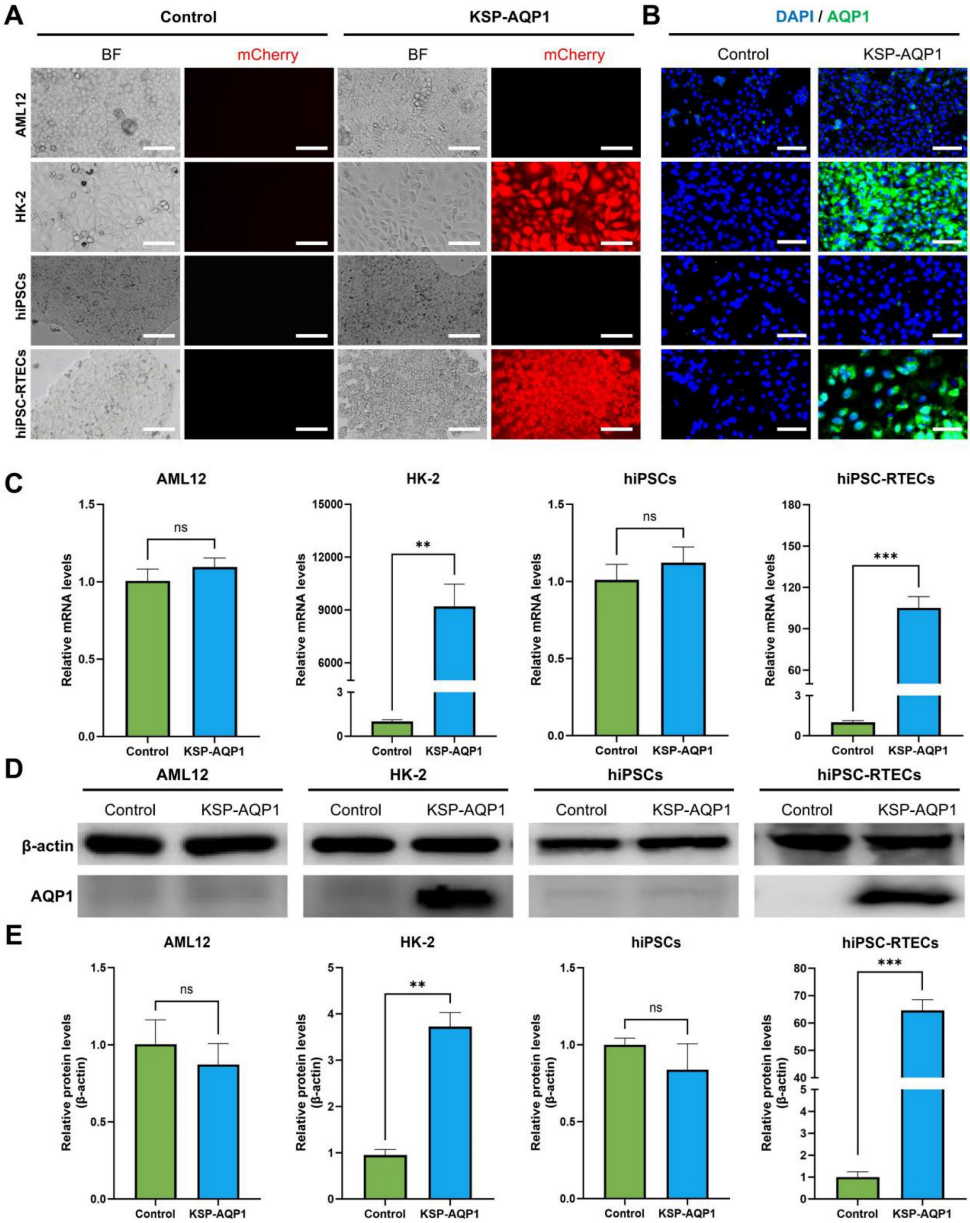
** KSP promoter-mediated specific expression of AQP1.** (**A**) The mCherry expression in each type of cell line before and after KSP-AQP1 transduction. BF, bright field; Scale bar, 100 μm. (**B**) IFA to detect the expression of AQP1. (**C**) RT-PCR to detect the expression level of AQP1 mRNA. (**D**) Western blot to analyze the level of AQP1 protein in different cells. (**E**) Quantitative analysis the relative level of AQP1 protein by western blot. **p < 0.01, ***p < 0.001, and ^ns^*p* > 0.05; *n* = 3.

To identify the specificity of KSP-AQP1, various cell lines including KSP-positive cell lines (HK-2 and hiPSC-RTECs) and KSP-negative cell lines (AML12 and hiPSCs) were tested. The presence of mCherry in KSP-AQP1 allowed us to visualize the expression directly by detecting red fluorescence. As illustrated in **Figure 3A**, HK-2 cells and hiPSC-RTECs exhibited pronounced red fluorescence, whereas KSP-negative cells displayed no significant red fluorescence after KSP-AQP1 transduction. These findings indicate the high specificity of KSP-AQP1. To further validate the specific overexpression of AQP1 in KSP-positive cells, IFA targeting AQP1 was conducted (**Figure 3B**). As expected, intense fluorescence was detected in HK-2 and hiPSC-RTECs specifically after KSP-AQP1 transduction. In addition, RT-PCR and western blot were also used to analyze the AQP1 mRNA and protein expression levels in both KSP-positive and KSP-negative cells. As shown in **Figure 3C-D**, KSP-positive HK-2 and hiPSC-RTECs exhibited significantly higher AQP1 expression than KSP-negative AML12 cells and hiPSCs.

### DWI-MRI tracking of the differentiation of hiPSCs into RTECs *in vitro*

**Figure 4:**
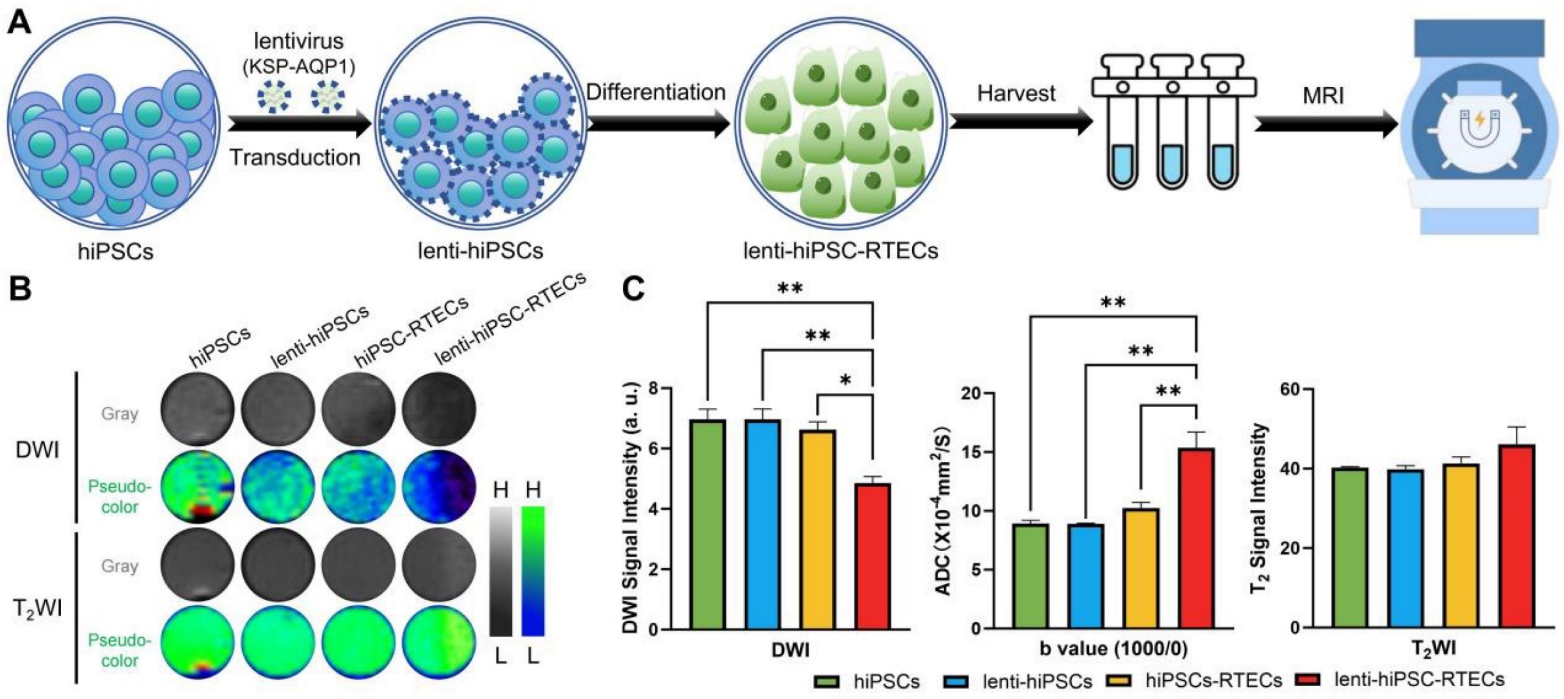
** KSP-AQP1-mediated specific DWI contrast after the differentiation of hiPSCs into RTECs.** (**A**) Schematic illustration of *in vitro* MRI. (**B**) DWI (*b* = 1000) images and T_2_WI signal maps of hiPSCs, lenti-hiPSCs, hiPSC-RTECs and lenti-hiPSC-RTECs, where L and H denote low SI and high SI, respectively. (**C**) Quantitative analysis of DWI SI, ADC*_b_*_=1000/0_ values, and T_2_WI SI in the target regions. *p < 0.05 and **p < 0.01; *n* = 3.

*In vitro* MRI of cells was performed following the workflow illustrated in **Figure 4A**. After the cells were collected, the DWI and T_2_WI of cells were conducted, with the results presented in **Figure 4B-C**. In terms of DWI SI, there were no significant differences between the hiPSCs, lenti-hiPSCs, and hiPSC-RTECs groups were detected. However, a significant decreased DWI SI was identified in lenti-hiPSC-RTECs, whereas no apparent change was detected in T_2_WI SI among the four cell groups. After quantitative analysis, lenti-hiPSC-RTECs exhibited a notable decrease in DWI SI by approximately 30.3% and an increase in ADC value by approximately 70.3% when compared with lenti-hiPSCs, and a decrease in DWI SI by approximately 26.6% and an increase in ADC value by approximately 50.4% when compared with hiPSC-RTECs. In addition, the SI of T_2_WI demonstrated no significant differences among the four groups. These results demonstrate that KSP-AQP1 can effectively track the differentiation of hiPSCs into RTECs via DWI-MRI at the cellular level.

### Tracking the differentiation of hiPSCs in I/R-AKI rats

**Figure 5:**
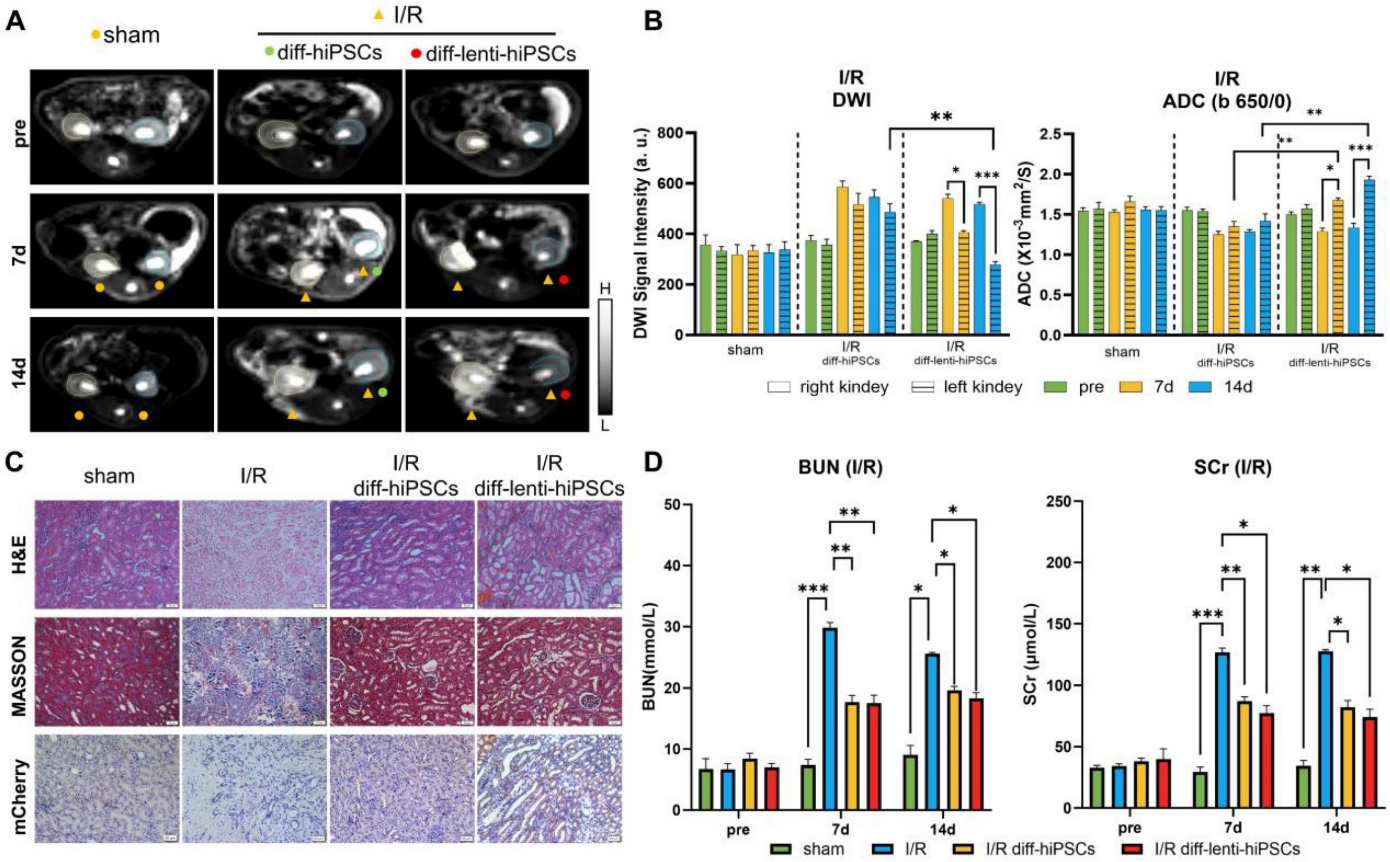
** DWI tracking the differentiation of hiPSCs in I/R-AKI rats**. (**A**) DWI images (*b* = 650) in different groups, where L and H denote low SI and high SI, respectively; pre, before I/R surgery. (**B**) Quantitative analysis of the DWI SI (*b*=650) and ADC*_b_*_=650/0_ values of ROI in Figure 5A. (**C**) H&E, Masson's trichrome staining, and IHC of kidney in different groups. Scale bar, 50 μm. (**D**) Serum concentrations of BUN and SCr before I/R and 7 and 14 days post-treatment in different groups. *p < 0.05, **p < 0.01, and ***p < 0.001; *n* = 3.

To evaluate the efficiency of KSP-AQP1 in tracking the differentiation of hiPSCs into RTECs *in vivo*, a rat model of renal I/R-AKI was established. Following the induction of I/R-AKI, diff-hiPSCs or diff-lenti-hiPSCs transplantation was performed, and DWI was performed to monitor cellular differentiation. As presented in **Figure 5A-B.** In the sham group, no significant differences in DWI SI or ADC*_b_*_=650/0_ values were observed between left and right kidneys across all time points, indicating stable renal function and absence of injury. In I/R + diff-hiPSCs group, the DWI SI showed no significant inter-kidney differences at 7 days (I/R: 589.59 ± 40.05 vs. I/R + diff-hiPSCs: 517.17 ± 74.20; *p* > 0.05) and 14 days (I/R: 545.4 ± 49.68 vs. I/R + diff-hiPSCs: 486.95 ± 56.13; *p* > 0.05). Correspondingly, no significant differences in ADC*_b_*_=650/0_ were detected between the I/R kidney and I/R+diff-hiPSCs-treated kidney at both 7 and 14 days. For the group of I/R + diff-lenti-hiPSCs, a significant DWI SI reduction was observed at day 7 when compared with the control I/R kidney (I/R: 541.27 ± 27.55 vs. I/R + diff-lenti-hiPSCs: 406.19 ± 12.22; *p* < 0.05), and further intensified at day 14 (I/R: 518.56 ± 11.20 vs. I/R + diff-lenti-hiPSCs: 279.18 ± 18.47; *p* < 0.001). The ADC*_b_*_=650/0_ of I/R+diff-hiPSCs-treated kidney showed a significant increase at day 7 by approximately 29.5%, and persisting at day 14 by approximately 45.1% when compared with the control I/R kidney. Besides analyzing the difference between I/R kidney and stem cells-treated kidney, we further compared the difference between diff-hiPSCs and diff-lenti-hiPSCs treated kidneys. Significant differences in DWI SI and ADC values were observed among the two treatments at 14 days, indicating the overexpression of AQP1 resulted in the significant change of DWI SI and ADC values. In addition to calculating the ADC values using *b*=650 and *b*=0, two high *b* values (*b*=1000 and *b*=650) were also used here to reduce the impacts of perfusion effect *in vivo*, and ADC*_b_*_=1000/650_ values were showed in **Figure S3**. Specifically, significant increased ADC*_b_*_=1000/650_ values were detected in diff-lenti-hiPSCs-treated kidney when compared with the control I/R kidney, or with diff-hiPSCs-treated kidney at both 7 and 14 days.

Moreover, the levels of BUN and SCr in serum, which reflect the kidney function, were detected before I/R, and 7 and 14 days after I/R with different treatments (**Figure 5D**). The BUN and SCr levels were significantly higher in the I/R group than those in the sham group, which proved that I/R injury successfully induced renal dysfunction in rats. Compared with those in the I/R group, BUN and SCr levels were significantly decreased in both diff-hiPSCs and diff-lenti-hiPSCs groups. However, no significant difference between these two groups was detected, indicating that hiPSCs play a role in the restoration of renal function caused by I/R injury. Furthermore, a histological analysis of the kidney was performed at 14 days after the I/R injury (**Figure 5C**). In contrast to sham group, the H&E staining revealed significant damage to the tubular cells in I/R characterized by diffuse shedding, dilation, and cast formation. The analysis of cell density revealed that I/R markedly decreased tissue cell density. However, after the treatment with hiPSCs, there was no significant difference in cell density was observed within hiPSCs, lenti-hiPSCs, and normal controls (**Figure S4**). Masson's trichrome staining demonstrated a significant increase in fibrosis following I/R injury, whereas treatment with diff-hiPSCs or diff-lenti-hiPSCs decreased the degree of fibrosis markedly. The renal tubular injury scores, as presented in **Figure S5**, demonstrated a significant reduction in the hiPSCs-treated group compared with the I/R group. These findings further corroborate the observation that hiPSCs treatment ameliorates the extent of injury caused by I/R.

To confirm that the differentiation of hiPSCs into RTECs in I/R kidney, we used the KSP-regulated mCherry, a non-endogenous gene co-expressed with AQP1, and visualized it via IHC (**Figure 5C**). The IHC results demonstrated significant expression of mCherry in kidney tissue following treatment with diff-lenti-hiPSCs. This finding suggests that in the I/R diff-lenti-hiPSCs group, the KSP promoter was successfully activated, which is attributed to the differentiation of hiPSCs into RTECs.

### Tracking the differentiation of hiPSCs in cisplatin-induced AKI rats

**Figure 6:**
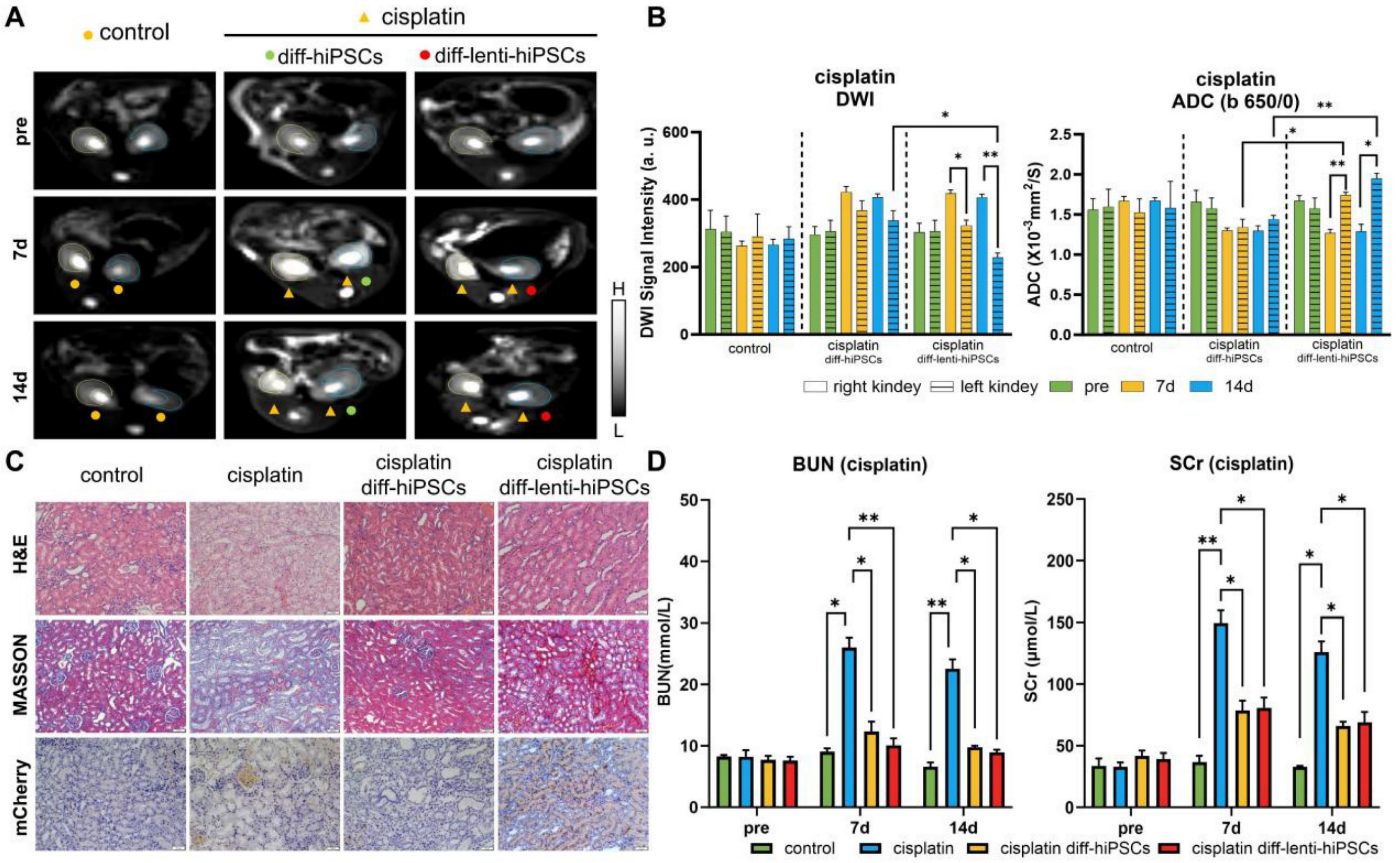
** DWI tracking the differentiation of hiPSCs in Cisplatin-AKI rats**. (**A**) DWI maps (*b* = 650) in different groups, where L and H denote low SI and high SI, respectively; pre, before cisplatin induction. (**B**) Quantitative analysis of the DWI SI (*b* = 650) and ADC*_b_*_=650_ values in Figure 6A. (**C**) H&E, Masson's trichrome staining, and IHC of kidneys in different treatments. Scale bar, 50 μm. (**D**) Serum concentrations of BUN and SCr before cisplatin induction and 7 days and 14 days post-treatment. *p < 0.05 and **p < 0.01; *n* = 3.

In addition to I/R, drug toxicity also frequently results in AKI in clinical practices. Consequently, cisplatin, an extensively used chemotherapeutic agent, was used here as an alternative model to induce AKI to facilitate a more comprehensive investigation of the role of KSP-AQP1 in monitoring the differentiation of hiPSCs. As demonstrated in **Figure 6A-B**, no significant differences in DWI SI or ADC*_b_*_=650/0_ values were observed between left/right kidneys across all time points in the control group. In cisplatin-treated kidneys, the DWI SI increased at both 7 and 14 days, and the ADC*_b_*_=650/0_ values were decreased at 7 and 14 days after cisplatin treatment. However, after the treatment with diff-lenti-hiPSCs, the DWI SI reduced significantly at 7 and 14 days post-treatment, and the ADC*_b_*_=650/0_ values increased at 7 and 14 days correspondingly. In addition to examining the differences between cisplatin-induced AKI kidneys and those treated with stem cells, we also investigated the differences between kidneys treated with diff-hiPSCs and those treated with diff-lenti-hiPSCs. Notable differences in DWI SI and ADC values were also observed between the two treatment groups at 14 days post-treatment. These findings suggested that the overexpression of AQP1 significantly alters DWI SI and ADC values. Besides calculating the ADC value using *b*=650 and *b*=0, two high *b* values were also used here to calculate the ADC value to reduce the perfusion effect *in vivo*, and the ADC*_b_*_=1000/650_ values were showed in **Figure S6**. Similar to I/R model, the diff-lenti-hiPSCs treatment significantly promoted the ADC*_b_*_=1000/650_ values at 7 and 14 days post-treatment when compared with diff-hiPSCs treatment.

To further confirm the induction of AKI by cisplatin and the therapeutic efficacy of hiPSCs, both pathological and serum biochemical analyses of renal function were performed, analogous to the I/R-AKI model. As illustrated in **Figure 6C-D**, the H&E staining revealed significant necrosis of renal tubular cells following cisplatin induction. In contrast, after the treatment with hiPSCs, the extent of injury attenuated markedly. The cell density analysis results revealed that cisplatin markedly decreased tissue cell density. However, after the treatment with hiPSCs, no significant difference in cell density was observed between diff-hiPSCs and diff-lenti-hiPSCs groups (**Figure S7**). The Masson's trichrome staining results also suggested a significant increase in collagen fiber deposition after cisplatin treatment. However, after the treatment with diff-hiPSCs or diff-lenti-hiPSCs, the renal tubular injury score was markedly decreased (**Figure S8**). To confirm the differentiation of hiPSCs into RTECs, IHC was also used to detect the KSP-controlled exogenous mCherry protein, which revealed a pronounced expression of mCherry in the kidney. In addition, BUN and SCr levels were also measured and showed a trend consistent with the I/R-induced AKI model.

## Discussion

The injury to renal parenchymal cells due to AKI often involves apoptosis and necrosis of the RTECs and can thus lead to several nephrotic syndromes. Therefore, it is necessary to restore the proper function of the renal parenchyma by regeneration of the damaged tubular epithelium. Of various therapeutic options, stem cells appear to provide a promising future owing to their regenerative potential and pluripotent differentiation features. Although monitoring the differentiation of stem cells is crucial for therapeutic applications, predicting the extent of stem cell differentiation in damaged regions poses significant challenges because of the inherent variability in the microenvironment of injury among individuals.

MRI has been extensively used for *in vivo* stem cell tracking because of its noninvasive, safety profile, and high tissue penetration depth. Currently, many stem cell labeling technologies are available to enable MRI-based *in vivo* tracking, such as using magnetic nanoparticles for stem cell labeling. Nevertheless, these technologies using exogenous probes are limited to tracking the stem cells, exhibiting some constraints in monitoring their differentiation. This limitation arises because differentiation tracking requires the exogenous probes to exhibit different MRI signals post-differentiation, a criterion that is often difficult to achieve. Moreover, the intrinsic proliferation nature of stem cells results in a continuous reduction in the content of exogenous probes with each cell division *in vivo*, thereby rendering them inadequate for differentiation tracking. Conversely, endogenous MRI contrast agents, which depend on stable gene expression, present a more effective alternative to exogenous labeling methods for their controllable stable expression. In this study, we successfully developed an endogenous MRI reporter gene system using a KSP promoter to drive a downstream MRI reporter gene, enabling the *in vivo* tracking of differentiation processes.

The selection of an appropriate gene delivery vector is among the first crucial considerations. Currently, various strategies have been developed for mammalian cells; liposomes, plasmids, adenoviral vectors, and lentiviral vectors are the most common. Of particular note, lentivirus-based delivery strategies have major advantages: they allow stable integration into the host cell genome, can mediate long-term regulation of gene expression, and have, to date, not been associated with major toxicity issues. To monitor the differentiation process, the MRI signal in the hiPSCs thus must be turned off, only to be switched back upon differentiation, which is necessary for the differentiation process to be tracked. The KSP promoter was used to turn this signal off, and it is known to be inactive in hiPSCs but activated upon their differentiation into RTECs, allowing the expression of downstream genes. To analyze the specificity, we tested AML12, HK-2, hiPSCs, and hiPSC-RTECs. The findings indicated that the KSP promoter-based gene regulation strategy can selectively trigger the expression of downstream genes in RTECs, including hiPSC-RTECs. This result underscores the specificity of the KSP promoter.

Selection of MRI reporter genes is often challenging because many have been documented, but such reporters often suffer from limitations in sensitivity. For instance, ferritin-based MRI reporter genes often require additional iron sources. In this study, we chose aquaporins. Most obviously, at least 13 types of aquaporins are present within the human body 46, 47; they are mostly located in the brain and intestine 48. AQP1 is one of the first characterized water channel proteins and has been well studied compared with other aquaporins, with sufficiently complete biochemical knowledge of the water permeability mechanism and its biological effects. Several studies have revealed that AQP1 and AQP4 have superior water penetration potential and that their expression levels correlate better with DWI SI *in vivo*. Nonetheless, a parallel study revealed that overexpression of AQP1 leads to greater DWI enhancement than AQP4. Previous research has demonstrated that even a minimal proportion of cells, as low as 5%, exhibiting increased AQP1 expression can lead to significant changes in DWI signals and ADC values 49, 50. Consequently, we selected the AQP1 gene as the MRI reporter gene in our study. Our experimental results demonstrated that following the successful differentiation of hiPSCs into RTECs, there was a marked increase in AQP1 expression levels in the cells that were previously transduced with KSP-AQP1.

To facilitate non-invasive monitoring of water molecule movement, we employed a widely used clinical MRI scanning sequence-DWI. However, due to DWI's sensitivity to variables such as cell density, shape, size, and physical parameters during MRI scanning, careful control is essential throughout the study. In this study involving cellular MRI, we implemented measures to mitigate these effects by organizing four groups of samples, with a focus on hiPSC-RTECs. The primary theoretical distinction between the hiPSC-RTECs and the lenti-hiPSC-RTECs groups lies in the expression level of AQP1. For these cell samples, we initially eliminated dead cells and utilized cell precipitation as the scanning sample to prevent density variations due to suspension sedimentation. Furthermore, we stabilized the relative position of the sample and the coil, and altered the order of sample placement multiple times to minimize deviations in detection results caused by magnetic field heterogeneity. Moreover, the *b*-value is a crucial parameter in DWI, as it directly affects both sensitivity and the SNR. Compared to 3.0 T MRI, 7.0T MRI provides an improved SNR and gradient system, allowing a high *b*-value to sustain a high SNR. In this study, significant changes in DWI SI and ADC values were observed exclusively in lenti-hiPSC-RTECs, which exhibited elevated expression levels of AQP1. However, the SI of T_2_WI did not exhibit significant changes, further suggesting that the observed changes in DWI were attributable to variations in the expression level of AQP1.

Another potential limitation of AQP1-based DWI is the occurrence of negative contrast enhancement. Furthermore, lesions characterized by AQP1 overexpression may be indistinguishable from infectious lesions or regions exhibiting high ADC values. In addition, during the recovery phase of AKI, the DWI signal may resemble that of adjacent healthy renal tissue. Therefore, to elucidate the specific differentiation of stem cells in the context of AKI treatment, it is essential to undertake a longitudinal analysis of their differentiation processes. In this study, DWI scans were conducted at 7 and 14 days post-hiPSCs transplantation. The findings indicated that the DWI SI and ADC value of the KSP-AQP-pretransduced hiPSCs progressively changed following transplantation into AKI renal tissue. Following the transplantation of hiPSCs in both I/R and cisplatin-induced AKI models for 7 days, the group of rats that received diff-lenti-hiPSCs exhibited a significantly decreased DWI SI and a significantly increased ADC value compared with the group transplanted with hiPSCs alone. This observed difference was hypothesized to result from the increased expression of AQP1 following differentiation. At 14 days, changes in DWI signals and ADC values were also observed in the hiPSCs group. These changes may be attributed to the extensive differentiation of hiPSCs into RTECs, as aquaporins were frequently expressed in these cells. However, a significant difference was still identified between the diff-hiPSC and diff-lenti-hiPSCs groups. This further suggested that AQP1 overexpression post-differentiation can serve as a marker for tracking differentiation. In addition, to verify the differentiation of hiPSCs in AKI, an exogenous flag protein, mCherry, was identified using IHC. As mCherry was regulated by the KSP promoter, its expression serves as a direct indicator of KSP promoter activation and, consequently, as an indirect marker of hiPSCs differentiation into RTECs.

Totally, considering the advancements in genetic engineering, it is conceivable that AQP1-based reporters, when paired with distinct promoters, could be utilized to visualize specific biological process *in vivo* by altering the water motion through DWI. Furthermore, for the advancement of filter-exchange imaging in evaluating the permeability of cell membranes, the genetic reporter AQP1 enables the quantitative tracking of specific biological processes in a non-invasive manner.

## Conclusions

The findings indicate that the KSP promoter-mediated overexpression of AQP1 allows for noninvasive DWI visualization of hiPSCs differentiation into RTECs *in vivo*. Leveraging the extensive use of DWI and stimulated echo pulse sequences in clinical diagnostics, the AQP1-based MRI could smoothly translate from laboratory studies to clinical applications. This method is expected to provide brand-new perspectives and strategies for tracking differentiation during stem cell-based therapies.

## Supplementary Material

Supplementary methods, table, file, and figures.

## Abbreviations

AQP1: aquaporin 1
hiPSCs: human-induced pluripotent stem cells
RTECs: renal tubular epithelial cells
I/R: ischemia/reperfusion
AKI: acute kidney injury
SI: signal intensity
MRI: magnetic resonance imaging
T_2_WI: T_2_-weighted imaging
DWI: diffusion-weighted imaging
ADC: apparent diffusion coefficient
BUN: blood urea nitrogen
SCr: serum creatinine
PCR: polymerase chain reaction
RT-PCR: reverse transcription-polymerase chain reaction
IFA: immunofluorescence staining assay
PBS: Phosphate Buffered Saline
