# Supplementary material for: Noninvasive DWI tracking of hiPSCs differentiation into RTECs in AKI recovery via the KSP promoter-mediated AQP1 strategy
Source: Theranostics. 2025 Apr 9;15(11):5106–20. doi: 10.7150/thno.109826 (PMC12036887; doi:10.7150/thno.109826)
Supplement: Supplementary file 1 — Supplementary methods, table, file, and figures. [file thnov15p5106s1.pdf]

Supplementary Material

Noninvasive DWI tracking of hiPSCs differentiation into RTECs in AKI recovery via the KSP promoter–mediated AQP1 strategy

Contents:

Extend Method. Tubular Injury Scoring Criteria

Supplementary Table S1. The PCR primers used in this study

Supplementary File 1. AQP1 gene and KSP-cadherin promoter gene sequencing

Supplementary Figure S1. Schematic diagram of plasmid construction

Supplementary Figure S2. Schematic of the workflow for ROI drawing *in vivo* MRI.

Supplementary Figure S3. Quantitative analysis of the ADC values of ROI in I/R-AKI model.

Supplementary Figure S4. Quantitative analysis of cell density in the H&E-stained I/R-AKI kidney sections.

Supplementary Figure S5. Histological tubular injury score of kindey in I/R-AKI model.

Supplementary Figure S6. Quantitative analysis of the ADC values of ROI in cisplatin-induced AKI rats.

Supplementary Figure S7. Quantitative analysis of cell density in the H&E-stained kidney sections in cisplatin-induced AKI.

Supplementary Figure S8. Histological tubular injury score of kidney in cisplatin-induced AKI rat.

Extend Method

Tubular Injury Scoring Criteria

A score of 0 means no kidney injury. RTECs are intact, no cast formation, and renal tubules not dilated; kidneys are healthy. A score of 1-3 indicates mild injury. RTECs may have slight changes (e.g., mild swelling in < 30% of renal tubules), occasional minimal cast formation, or mild dilation (diameter < 50% increase). This mild injury may result from short-term, mild kidney stimuli, with only a slight impact on kidney function and potential recovery after injury factor removal. A score of 4-6 shows moderate injury. For example, moderate RTECs necrosis (like increased cytoplasmic vacuoles, partial brush border loss), moderate cast formation (2-5 casts per 10 high-power fields on average), and tubular dilation (50%-100% diameter increase). This reveals obvious kidney injury and some damage to tubular function. Further observation or treatment is needed to prevent worsening. A score of 7-9 implies severe injury. There may be extensive RTECs necrosis (> 70% of renal tubules affected), numerous cast formations (>5 casts per 10 high-power fields on average), and severe tubular dilation (diameter > 100% increase).

.

Table S1. The PCR primers used in this study

Primers	Sequence (Sense/Antisense)
OCT4	CTTCGCAAGCCCTCATTTC
	GAGAAGGCGAAATCCGAAG
Nanog	ATGCCTCACACGGAGACTGT
	CAGGGCTGTCCTGAATAAGC
CDH1	GAACGCATTGCCACATACAC
	GCACCTTCCATGACAGACCC
CK18	CGCATCGTCTTGCAGATCGAC
	GCTGAGACCAGTACTTGTCCAG
AQP1	CTATGCGTGCTGGCTACTACCG
	CCAAAGGACCGAGCAGGGTT
GAPDH	ACCCACTCCTCCACCTTTGAC
	CCACCACCCTGTTGCTGTAG
Plasmid	CCGAGTTCCTGGCCACGACC
	CTTGTACTCGGTCATGGTG

Supplementary File 1. AQP1 gene and KSP-cadherin promoter gene sequencing

ACAAATTACAAAAATTCAAAATTTTCGGGTTTATTACAGGGACAGCAGAGATCCAGTTT
GGTTAATTAAAAGTGGGAGCCAAGTCTGAACACACACACACACACACACACACACAC
ACACAGAAGTTATGTCTAAGCCCAGAGGGCCAGCAAGTGCTCATTGGGCTGTGTCAAG
GGGGCAGTGACAGACCAAGAGCTGCCCACCTCCTAGGGCTGGCAGTCACGGATGCTG
AGCAGATCTGGCTCTCCAAAGTCAATAAGTAACTTGGGGGGACTAGGCGGGGCCAGG
CCTGCTCCTGTGGGCCCCGGTGGCATTTTCCACTCCTGAGCAAGCACGGCCAGACCGC
CTACCTGCTCAAGTGTCCACCTTGCCTCGCCCCACCTAAGCCAAATTGCTAGCGTTTAA
ACGGGCCCTCTAGACTCGAGCGCCACCATGGTGAGCAAGGGCGAGGAGGATAACATG
GCCATCATCAAGGAGTTCATGCGCTTCAAGGTGCACATGGAGGGCTCCGTGAACGGCC
ACGAGTTCGAGATCGAGGGCGAGGGCGAGGGCCGCCCCTACGAGGGCACCCAGACCG
CCAAGCTGAAGGTGACCAAGGGTGGCCCCCTGCCCTTCGCCTGGGACATCCTGTCCCC
TCAGTTCATGTACGGCTCCAAGGCCTACGTGAAGCACCCCGCCGACATCCCCGACTAC
TTGAAGCTGTCCTTCCCCGAGGGCTTCAAGTGGGAGCGCGTGATGAACTTCGAGGAC
GGCGGCGTGGTGACCGTGACCCAGGACTCCTCCCTGCAGGACGGCGAGTTCATCTACA
AGGTGAAGCTGCGCGGCACCAACTTCCCCTCCGACGGCCCCGTAATGCAGAAGAAGA
CCATGGGCTGGGAGGCCTCCTCCGAGCGGATGTACCCCGAGGACGGCGCCCTGAAGG
GCGAGATCAAGCAGAGGCTGAAGCTGAAGGACGGCGGCCACTACGACGCTGAGGTCA
AGACCACCTACAAGGCCAAGAAGCCCGTGCAGCTGCCCGGCGCCTACAACGTCAACA
TCAAGTTGGACATCACCTCCCACAACGAGGACTACACCATCGTGGAACAGTACGAACG
CGCCGAGGGCCGCCACTCCACCGGCGGCATGGACGAGCTGTACAAGGGCAGCGGCGA
GGGCAGAGGAAGTCTTCTAACATGCGGTGACGTGGAGGAGAATCCCGGCCCTATGGCC
AGCGAGTTCAAGAAGAAGCTCTTCTGGAGGGCAGTGGTGGCCGAGTTCCTGGCCACG
ACCCTCTTTGTCTTCATCAGCATCGGTTCTGCCCTGGGCTTCAAATACCCGGTGGGGAA
CAACCAGACGGCGGTCCAGGACAACGTGAAGGTGTCGCTGGCCTTCGGGCTGAGCAT
CGCCACGCTGGCGCAGAGTGTGGGCCACATCAGCGGCGCCCACCTCAACCCGGCTGT
CACACTGGGGCTGCTGCTCAGCTGCCAGATCAGCATCTTCCGTGCCCTCATGTACATCA
TCGCCCAGTGCGTGGGGGCCATCGTCGCCACCGCCATCCTCTCAGGCATCACCTCCTC
CCTGACTGGGAACTCGCTTGGCCGCAATGACCTGGCTGATGGTGTGAACTCGGGCCAG
GGCCTGGGCATCGAGATCATCGGGACCCTCCAGCTGGTGCTATGCGTGCTGGCTACTAC
CGACCGGAGGCGCCGTGACCTTGGTGGCTCAGCCCCCCTTGCCATCGGCCTCTCTGTA
GCCCTTGGACACCTCCTGGCTATTGACTACACTGGCTGTGGGATTAACCCTGCTCGGTC
CTTTGGCTCCGCGGTGATCACACACAACTTCAGCAACCACTGGATTTTCTGGGTGGGG
CCATTCATCGGGGGAGCCCTGGCTGTACTCATCTACGACTTCATCCTGGCCCCACGCAG
CAGTGACCTCACAGACCGCGTGAAGGTGTGGACCAGCGGCCAGGTGGAGGAGTATGA
CCTGGATGCCGACGACATCAACTCCAGGGTGGAGATGAAGCCCAAATAGGAATTCCTG
TGGAATGTGTGTCAGTTAGGGTGTGGAAAGTCCCCAGGCTCCCCAGCAGGCAGAAGTA
TGCAAAGCATGCATCTCAATTAGTCAGCAACCAGGTGTGGAAAGTCCCCAGGCTCCCC
AGCAGGCAGAAGTATGCAAAGCATGCATCTCAATTAGTCAGCAACCATAGTCCCGCCC
CTAACTCCGCCCATCCCGCCCCTAACTCCGCCCAGTTCCGCCCATTCTCCGCCCCATGG
CTGACTAATTTTTTTTATTTATGCAGAGGCCGAGGCCGCCTCTGCCTCTGAGCTATTCCA
GAAGTAGTGAGGAGGCTTTTTTGGAGGCCTAGGCTTTTGCAAAAAG

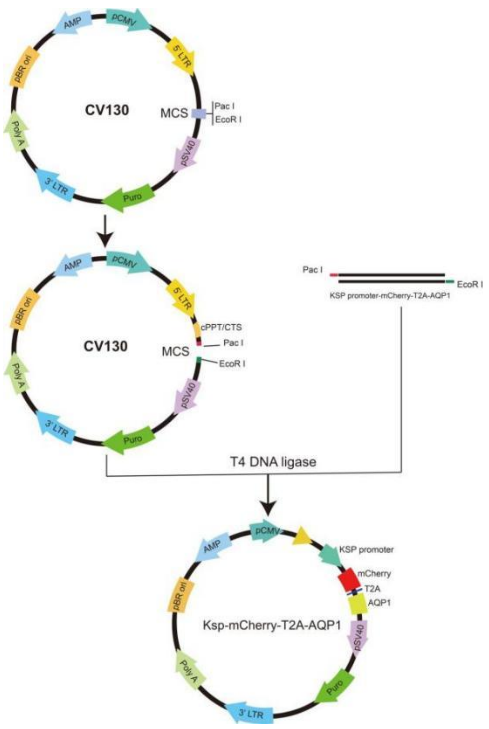

Figure S1. Schematic diagram of plasmid construction

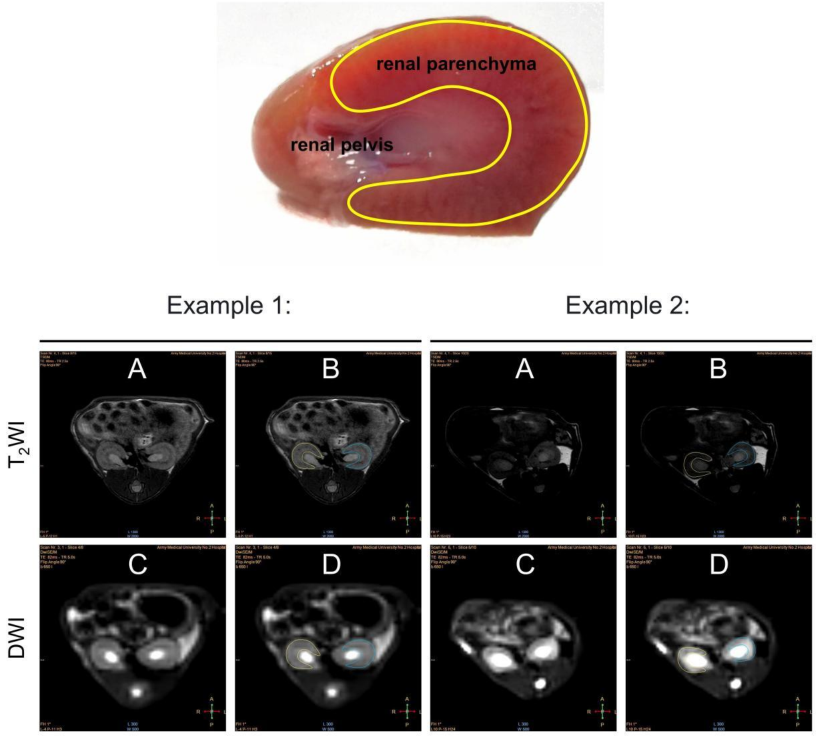

Figure S2. Schematic of the workflow for ROI drawing *in vivo* MRI. Initially, the ROI was delineated on the renal parenchyma using a T_2WI, as illustrated in Figure B. This ROI was then transferred onto the DWI shown in Figure C, thereby establishing the ROI on the DWI (Figure D).

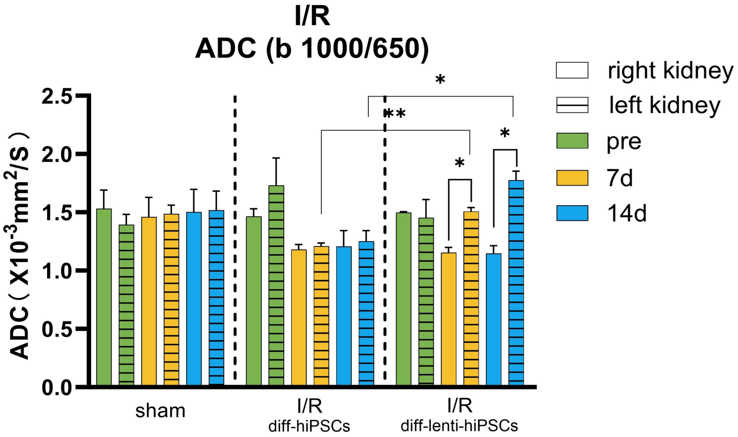

Figure S3. Quantitative analysis of the ADC values of ROI in I/R-AKI model. *$p < 0.05$ and **$p < 0.01$; n = 3

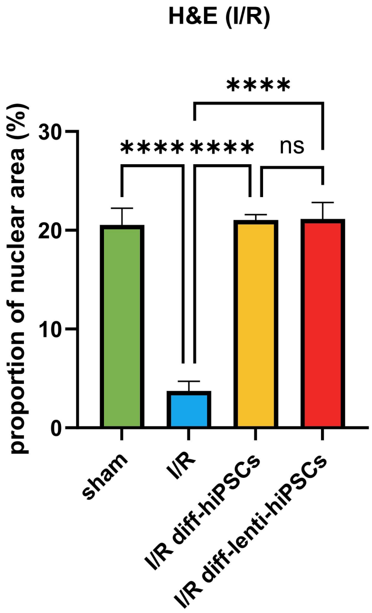

Figure S4. Quantitative analysis of cell density in the H&E-stained I/R-AKI kidney sections. $^{ns}p > 0.05$ and ****$p < 0.0001$; n = 3.

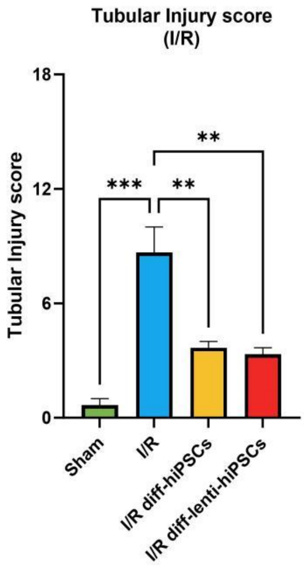

Figure S5. Histological tubular injury score of kindey in I/R-AKI model. **$p < 0.01$ and ***$p < 0.001$; n = 3.

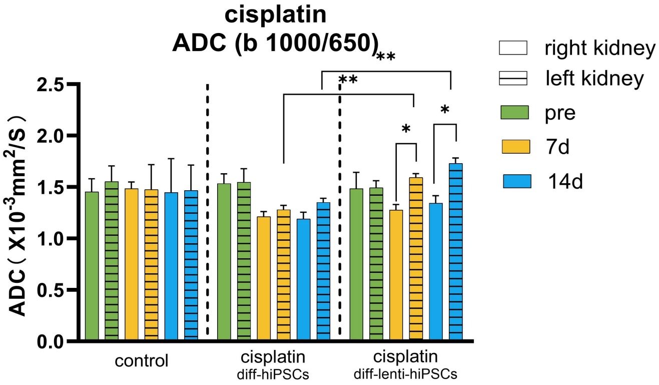

Figure S6. Quantitative analysis of the ADC values of ROI in cisplatin-induced AKI rats. *$p < 0.05$ and **$p < 0.01$; n = 3.

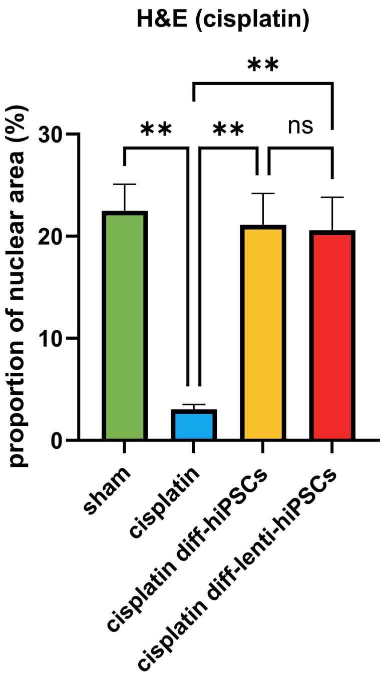

Figure S7. Quantitative analysis of cell density in the H&E-stained kidney sections in cisplatin-induced AKI. $^{ns}p > 0.05$ and $^{**}p < 0.01$; n = 3.

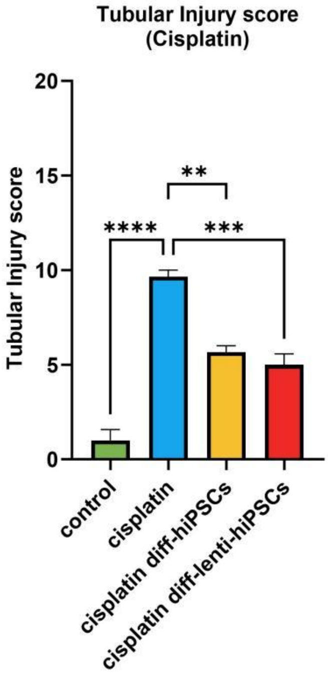

Figure S8. Histological tubular injury score of kidney in cisplatin-induced AKI rat. $^{**}p < 0.01$, $^{***}p < 0.001$, and $^{****}p < 0.0001$; n = 3.
